# Research progress on the effects of implant-supported overdentures on residual ridge remodelling in edentulous jaws: a narrative review

**DOI:** 10.2340/aos.v85.47067

**Published:** 2026-10-28

**Authors:** Nana Fan, Liqin Gong, Liqin Tang, Qiang Xue

**Affiliations:** Department of Prosthodontics, Wuxi Stomatology Hospital, Wuxi City, Jiangsu Province, China

**Keywords:** Edentulism, dental implantation, implant-supported overdenture, alveolar bone loss, bone remodelling

## Abstract

Residual ridge resorption (RRR) following tooth loss is a continuous and irreversible process that limits the effectiveness of conventional complete dentures and compromises masticatory function and quality of life. Implant-supported overdentures (implant overdentures [IODs]) can reconstruct physiological stress distribution, regulate inflammatory molecular signalling and optimise bone remodelling through combined biological and mechanical pathways, thereby helping to slow RRR. This review systematically summarises the biological mechanisms of RRR, including inflammatory mediators, the receptor activator of nuclear factor-kappa B ligand/osteoprotegerin pathway and systemic factors; the mechanical mechanisms, including Wolff’s law and stress distribution patterns; the protective effects of IODs on residual ridges; key determinants of their bone-preserving efficacy, including implant-, attachment-, occlusion- and patient-related factors; current controversies; and advances in digital applications. Regarding attachment systems, current evidence from randomised controlled trials, long-term cohort studies and meta-analyses indicates that ball, Locator and bar attachments demonstrate broadly comparable peri-implant marginal bone outcomes, although Locator attachments may be associated with lower posterior RRR over 10 years, whereas bar attachments may offer superior crestal bone stability in specific clinical scenarios. No single attachment system demonstrates a clinically meaningful, reproducible advantage in bone preservation, and selection should be individualised based on anatomical, functional and maintenance considerations. Current evidence remains limited by heterogeneous study designs, variable follow-up periods and an overreliance on finite element analyses, underscoring the need for standardised long-term clinical trials with harmonised outcome measures. Evidence from recent high-quality studies over the past 5 years is integrated to propose stratified clinical recommendations according to bone volume, systemic status and economic factors. Digital technologies, including computer-guided surgery, digital impressions and occlusal analysis, further enhance precision and predictability of bone-preserving outcomes. This review provides a theoretical basis and clinical reference for optimising rehabilitation protocols, preserving residual ridges over the long term and improving prosthetic prognosis in patients with edentulism.

## Introduction

Tooth loss represents the terminal stage of dentition defects and is particularly common among older adults. Following the loss of natural teeth, the alveolar ridge loses physiological occlusal stimulation and immediately enters a continuous resorptive process characterised by reduced bone height, decreased ridge width, lower bone density and flattening of the ridge crest. This process progresses at an estimated annual rate of approximately 0.5 mm in the maxilla and up to 1.0 mm in the mandible. These changes may ultimately lead to insufficient prosthetic space, poor retention and stability of conventional complete dentures, mucosal soreness, reduced masticatory efficiency and facial collapse, thereby severely impairing oral function and psychosocial health [1–3].

An implant-supported overdenture is a minimally invasive rehabilitative approach for patients with edentulous jaws. In this procedure, 2–4 implants are placed into the edentulous alveolar bone to provide retention and support, and attachments are used to stabilise the removable prosthesis. This approach preserves the convenience of removable complete dentures while markedly improving retention, support and function. Because satisfactory retention and support can be achieved with only 2–4 implants, implant-supported overdentures (implant overdentures [IODs]) are considered a minimally invasive, economical and reliable option. The McGill and York consensus statements established the two-implant mandibular overdenture as the preferred standard of care for patients with an edentulous mandible [4, 5].

In recent years, advances in imaging-based measurements, finite element analysis and long-term clinical cohort studies have made the effects of IODs on residual ridge stress distribution, bone metabolism and remodelling patterns an important research focus. Unlike previous reviews [6], this article focuses on the biomechanical synergy by which IODs modulate residual ridge remodelling, integrates and critically evaluates recent high-quality evidence from the past 5 years (particularly emphasising evidence published after 2021), critically interprets current controversies and proposes stratified clinical recommendations according to bone volume, systemic status and economic factors. A structured search strategy was developed in accordance with the Scale for the Assessment of Narrative Review Articles guidelines. PubMed, Embase and Web of Science were searched for English-language articles published up to 2026, using combinations of keywords such as ‘implant-supported overdenture’, ‘residual ridge resorption’ and ‘bone remodelling’. The inclusion criteria prioritised randomised controlled trials (RCTs), prospective cohort studies and finite element analyses, whereas case reports and non-peer-reviewed articles were excluded. The Joanna Briggs Institute critical appraisal tools were applied to assess the methodological quality of the included studies [7]. The aim was to avoid repetitive narrative review, strengthen mechanistic interpretation and enhance clinical guidance [8]. Against this background and in light of the remaining gaps in the literature, this review narratively and systematically summarises research progress on the influence of IOD rehabilitation on residual ridges in edentulous jaws and provides a reference for clinical practice and future research.

## Biological and mechanical mechanisms of residual ridge resorption

Residual ridge resorption (RRR) refers to the continuous loss of alveolar bone volume and changes in ridge morphology following tooth loss, resulting from the loss of physiological stimulation from natural teeth, abnormal mechanical loading and changes in the local microenvironment. It is a pathological remodelling process jointly mediated by local microenvironmental alterations, mechanical loading and systemic factors. The core mechanism is the uncoupling of osteogenesis and osteoclast-mediated resorption: bone resorption becomes substantially greater than bone formation, resulting in progressive loss of bone volume and ridge atrophy.

During the onset and progression of RRR, biological dysregulation and mechanical overload do not act independently; rather, they interact with and amplify each other, jointly causing bone metabolic imbalance and structural destruction. Therefore, a single mechanism cannot fully explain the complexity of persistent RRR [9, 10].

### Biological mechanisms

Natural teeth transmit occlusal forces to the alveolar bone through the periodontal ligament and convert them into physiological mechanical signals, which are essential for maintaining osteoblast activity. Osteocytes act as the primary mechanosensors embedded within the bone matrix, detecting mechanical strain and fluid shear stress. Under physiological loading, osteocytes activate the canonical Wnt/β-catenin signalling pathway and downregulate the Wnt inhibitor sclerostin, thereby promoting osteogenesis. Following tooth loss, this physiological stimulation disappears, leading to sclerostin upregulation and subsequent inhibition of Wnt signalling; consequently, osteogenic capacity declines, osteoclastic activity increases and bone resorption remains persistently elevated. Although these mechanistic insights are derived primarily from general bone biology rather than IOD-specific investigations, extrapolation to the edentulous ridge is biologically plausible and consistent with observed clinical patterns of RRR [11].

At the same time, edentulous areas are chronically exposed to denture-base compression, plaque accumulation and mucosal inflammation. These stimuli can induce high expression of pro-inflammatory cytokines, including tumour necrosis factor-alpha (TNF-alpha), interleukin (IL)-1 beta and IL-6. Extrapolating from general alveolar bone remodelling and orthodontic tooth movement models, it is understood that these cytokines directly activate the receptor activator of nuclear factor-kappa B ligand/osteoprotegerin (RANKL/OPG) signalling axis, promote osteoclast differentiation and survival, inhibit OPG synthesis, disrupt the RANKL/OPG balance and aggravate bone metabolic dysregulation [12, 13]. Therefore, residual ridge remodelling is a multifactorial process driven by the synergistic disruption of both the RANKL/OPG inflammatory axis and the Wnt/β-catenin mechanobiological pathway.

Systemic factors accelerate bone resorption by regulating bone metabolism and local blood supply, and their molecular associations are well established. First, ageing is accompanied by declining osteoblast function and reduced bone regenerative capacity. Second, oestrogen deficiency after menopause directly upregulates RANKL expression and promotes osteoclastogenesis. Third, osteoporosis (T-score: < −2.5) reduces bone mass and compromises bone microarchitecture, increasing susceptibility to bone resorption. Fourth, diabetes causes microcirculatory impairment and chronic low-grade inflammation, thereby compromising bone healing. Fifth, smoking damages vascularisation and immune function and interferes with bone metabolism and tissue repair [14–16].

### Mechanical mechanisms

The rate and morphology of RRR are highly dependent on the pathway of occlusal force transmission and the pattern of stress distribution. The morphology and internal structure of alveolar bone adapt to mechanical loading, a principle known as Wolff’s law [17, 18].

Conventional complete dentures are supported mainly by the mucosa. Occlusal forces are transmitted directly through the denture base to the residual ridge mucosa and underlying bone, which can generate stress concentration, localised high pressure and shear overload [19]. Quantitative biomechanical data indicate that peak mucosal stress under conventional complete dentures may exceed 200 kPa. Excessive compressive stress may cause local ischaemia and microcirculatory disturbance, thereby inducing pressure-related bone resorption. Shear stress may further lead to trabecular microdamage and disordered remodelling, accelerating loss of bone height and width.

### Biomechanical crosstalk in residual ridge remodelling

Although biological and mechanical mechanisms are categorised separately for clarity, they operate as a highly integrated biomechanical continuum during residual ridge remodelling. Mechanical forces dictate the cellular biological signalling cascade: the physiological mechanical loading restored by IODs is sensed by osteocytes, which translate mechanical strain into biological signals by downregulating sclerostin and activating the Wnt/β-catenin osteogenic pathway. Concurrently, the elimination of pathological mucosal overload (shear and excessive compressive stress) reduces local microcirculatory ischaemia and the subsequent release of pro-inflammatory cytokines (e.g. TNF-alpha and IL-6), thereby suppressing the RANKL/OPG axis and decreasing osteoclastogenesis. Ultimately, it is this synergistic interaction – in which normalised mechanical stress actively corrects inflammatory–molecular dysregulation – that shifts the remodelling balance from destructive resorption towards functional bone preservation. It should be acknowledged that part of this mechanistic framework is extrapolated from general bone remodelling evidence and requires IOD-specific validation [11–13].

## Protective effects of implant-supported overdentures on residual ridges

### Significant reduction in the rate of bone resorption

IODs transmit the main occlusal forces through implants directly to the implant–bone interface. Biomechanical simulations and finite element analyses indicate that this pathway reduces peak mucosal stress by approximately 60%–80%. This mechanism decreases stress concentration and shear loading, produces a more uniform stress distribution closer to the physiological state and guides residual ridge remodelling towards a functional, preservative pattern [20–22].

The quantitative evidence supporting these effects comes from diverse study designs and outcome measures, which cannot be directly compared as in a meta-analysis. Observational studies utilising cephalometric and panoramic radiographs demonstrate that, after 5 years of conventional complete denture use, overall residual ridge bone loss may reach 3.0–5.0 mm, with the mandibular anterior region being particularly affected [1]. In contrast, evaluations of peri-implant crestal bone levels using standardised intraoral periapical radiographs reveal that marginal bone loss around implants following IOD rehabilitation is approximately 0.5 mm over 5 years, with the annual rate stabilising at around 0.1 mm. Bone loss in non-implant regions is also markedly reduced. Radiographic comparative studies measuring posterior alveolar height indicate that the rate of mandibular posterior ridge resorption is only approximately half that observed with conventional complete dentures [22]. Long-term follow-up clinical trials utilising similar peri-implant measurements over 5–8 years have shown that marginal bone loss in IOD groups ranges from only 0.7 to 1.3 mm, indicating a substantially better outcome than conventional complete dentures [23, 24].

### Optimisation of bone remodelling patterns

IODs can simultaneously correct inflammatory signalling dysregulation and abnormal stress distribution, interrupting the vicious cycle of biological and mechanical injury. As a result, residual ridge remodelling may shift from passive destructive resorption towards functional and protective adaptation.

The physiological mechanical stimulation provided by implants can increase bone density around them and in the residual ridge, promote cortical thickening and optimisation of trabecular architecture and maintain the stability of ridge crest morphology [25]. Three-dimensional cone-beam computed tomography (CBCT) studies have confirmed that IOD rehabilitation significantly reduces loss of residual ridge height and width, thereby preserving an anatomical foundation for long-term prosthesis stability and future rehabilitation options [26, 27].

### Key factors influencing the bone-preserving effects of implant-supported overdentures

#### Implant-related factors

The number, diameter, length, position and loading protocol of implants all influence residual ridge stress distribution and bone remodelling. Although some earlier studies suggested no significant difference in bone-preserving effects between mandibular two-implant and four-IODs, a systematic review by Pham et al. [6] demonstrated that four-implant–retained overdentures reduce posterior RRR more effectively. Supported by this evidence, it was clarified that the superior stress distribution of four implants justifies their specific recommendation for patients with severe bone resorption, although the survival rate of two-mini-IODs is slightly lower [6].

A mandibular two-implant IOD placed in the canine region appears to provide the most favourable long-term maintenance of bone volume, whereas three-implant IODs may cause more extensive bone resorption and are therefore not recommended for routine clinical use. Because maxillary bone density is generally lower, four or more implants are recommended in the maxilla to improve stability. Increasing the number of implants in the anterior maxilla may reduce posterior maxillary bone resorption [28].

Narrow-diameter or mini implants are associated with less surgical trauma, but their long-term marginal bone loss is slightly greater than that of conventional-diameter implants. Adequate implant length improves primary stability and osseointegration efficiency and reduces the risk of bone resorption [29]. Proper control of the crown–root ratio and avoidance of excessive cantilever length can reduce lateral stress and protect the residual ridge. Immediate loading can restore function early and does not compromise implant survival or bone preservation, but the indications must be carefully selected [30].

### Selection of attachment systems

Commonly used attachment systems include Locator^®^, bar–clip, ball and magnetic attachments, each with distinct stress-transmission characteristics and specific clinical indications and benefits. These systems can be broadly categorised as non-splinted (ball, Locator and magnetic), which allow independent implant movement and provide varying degrees of stress interruption, and splinted (bar–clip), which rigidly connects implants through a cross-arch framework and offers maximum stability but limited stress buffering. Locator attachments provide resilient retention and a stress-breaking effect, resulting in the lowest stress peaks. Ball attachments are widely documented as a cost-effective, clinically robust option with excellent long-term survival and moderate stress distribution. Magnetic attachments are highly advantageous for patients with limited manual dexterity, providing favourable passive stress buffering without lateral torque. Bar–clip attachments function as rigid splinted structures with little buffering capacity and therefore produce the most evident stress concentration, yet they provide exceptional stability and cross-arch splinting and are highly beneficial in cases of compromised bone quality or extreme ridge atrophy.

Several three-dimensional finite element studies have shown that, under the same load, stress in implants and cortical bone with Locator attachments is approximately 30%–40% lower than that with bar–clip attachments, which may be more favourable for long-term stability [31–34]. Ball attachments transmit intermediate stress levels, higher than those of Locator attachments but lower than those of bar–clip systems, whereas magnetic attachments demonstrate the most uniform stress distribution due to their complete lack of lateral torque transmission. However, recent clinical evidence demonstrates that, in four-implant–retained overdentures, the spatial distribution of the implants significantly influences residual ridge remodelling and stress dissipation, independent of the specific attachment system utilised [30]. Locator attachments are widely used because of their stable retention, simple seating and optimal control of bone resorption. Bar–clip attachments provide strong stability and may be preferred for splinting in compromised bone. Therefore, clinical selection should be tailored to the individual’s anatomical and functional needs, prioritising stress buffering, uniform force transmission and appropriate implant distribution.

Beyond biomechanical predictions, clinical evidence regarding the influence of attachment design on peri-implant marginal bone loss has yielded nuanced and sometimes inconsistent findings. A 2024 systematic review and meta-analysis including 10 studies and 424 patients found no statistically significant difference in marginal bone loss between ball and Locator attachments, although the included studies exhibited substantial heterogeneity [35]. For ball versus bar attachments, a 5-year RCT reported 100% implant survival in both groups and no significant differences in marginal bone loss or peri-implant mucosal health, although the bar group experienced more frequent technical complications [36]. These findings were corroborated by a meta-analysis of RCTs demonstrating no significant influence of implant splinting (bar) versus non-splinting (ball/Locator) on marginal bone levels [37]. Notably, a 10-year retrospective analysis of 4-implant mandibular overdentures reported an apparent discordance with finite element predictions: Locator-retained prostheses exhibited significantly greater crestal bone loss (2.2 mm) than bar-retained prostheses (1.6 mm; *p* = 0.0001), despite Locator systems demonstrating lower stress peaks in computational models [38]. This discrepancy underscores the limitations of extrapolating finite element results to long-term clinical bone remodelling and highlights the need for cautious interpretation.

The influence of attachment systems on RRR in non-implant-bearing areas has been less extensively studied, but emerging long-term data suggest clinically relevant differences. The aforementioned 10-year retrospective analysis found that posterior mandibular ridge resorption was significantly lower in the Locator group (0.9 mm) than in the bar group (1.6 mm; *p* = 0.001) [38]. This observation is biologically plausible: non-splinted resilient attachments such as Locator allow a degree of implant movement and stress interruption, which may reduce the transmission of non-axial forces to the posterior denture-bearing mucosa and subsequently decrease pressure-induced resorption. Conversely, the rigid splinting provided by bar attachments may distribute occlusal forces more effectively across the implant framework but may also increase functional loading of the posterior residual ridge through the denture base. It is important to note that these RRR findings originate from a single retrospective study with a relatively small sample (*n* = 25), and further prospective validation is required before definitive clinical recommendations can be made.

Long-term clinical outcomes and maintenance requirements vary considerably among attachment systems and may indirectly affect bone preservation through the maintenance of optimal stress distribution over time. A 5-year RCT comparing ball, magnetic and Locator attachments in 69 patients found that all 3 systems functioned well, but magnetic attachments had the lowest maintenance burden (15 events) and the highest prosthetic success rate (82.6%), followed by Locator attachments (31 events, 78.2% success), whereas ball attachments required the highest number of maintenance interventions (195 events) [39]. For bar versus ball attachments, the 5-year RCT by Gotfredsen and Holm reported a higher annual frequency of technical complications in the bar group (1.0 per patient-year) than in the ball group (0.6 per patient-year) [36]. In the maxilla, a 1-year RCT of 4-IODs found that bar attachments were associated with significantly poorer peri-implant tissue health (higher plaque index, gingival index and bleeding on probing) and lower implant success rates than ball attachments, likely due to the greater difficulty of maintaining oral hygiene around bar frameworks [40]. Furthermore, progressive wear of attachment systems is a critical factor influencing long-term residual ridge preservation. As attachments undergo mechanical wear over time, their original retention capacity and stress-buffering properties degrade. This degradation alters the intended force transmission, often directing non-axial and unbuffered loads onto the implants and underlying alveolar bone. The resulting increase in peri-implant strain can accelerate localised RRR. Consequently, regular clinical evaluation of attachment wear and timely replacement of worn components are essential to maintain stable stress distribution and protect the residual ridge [41].

Critical interpretation of the available literature reveals substantial inconsistencies that must be acknowledged when evaluating the effects of attachment systems on bone outcomes. Two independent meta-analyses found no significant differences in marginal bone loss, prosthetic complications or implant survival between splinted (bar) and non-splinted (ball/Locator) attachment systems [37, 42], challenging the commonly held assumption that rigid bar splinting necessarily compromises peri-implant bone health. Several confounding factors may account for the discrepant findings across studies. First, implant number and distribution play a decisive role: a 10-year retrospective study demonstrated that the spatial arrangement of 4 Locator attachments significantly influenced residual ridge remodelling independently of attachment type [30]. Second, splinting versus non-splinting status interacts with implant number: two-implant bar overdentures and four-implant bar overdentures may produce fundamentally different stress environments, yet studies frequently pool these configurations. Third, jaw location modifies outcomes: maxillary bone is generally less dense than mandibular bone, and bar attachments may provide essential initial stability in the maxilla despite increased hygiene challenges [40]. Fourth, prosthetic design factors, including cantilever length, occlusal scheme and denture-base extension, can substantially alter load transmission regardless of attachment type. Fifth, loading protocols (immediate vs. conventional) and opposing dentition characteristics (natural teeth, implant-supported fixed prosthesis or complete denture) introduce additional variability. A systematic review of attachment system selection concluded that bar attachments provide the strongest retention, telescopic systems offer the highest patient satisfaction and the fewest mucosal changes and ball attachments are most suitable for limited inter-arch space and parallel implant placement [43]. Taken together, current evidence does not support the superiority of any single attachment system for preserving residual ridge or peri-implant bone. Rather, the optimal choice depends on a comprehensive assessment of implant position and distribution, bone quality, jaw location, manual dexterity, maintenance capacity and economic considerations.

Table 1 summarises the comparative evidence across ball, Locator and bar-clip attachment systems regarding stress distribution, marginal bone loss, RRR, long-term survival, maintenance requirements and clinical indications.

**Table 1:** Comparative evidence of ball, Locator and bar-clip attachment systems in implant-supported overdentures.

Comparison dimension	Ball	Locator	Bar-clip	Evidence level
Stress peak (FEA)	Intermediate	Lowest	Highest	Finite element analysis [31–34]
Marginal bone loss (clinical)	No significant difference vs. Locator [35]; no significant difference vs. bar [36, 37]	No significant difference vs. ball [35]; greater crestal loss than bar at 10 years (2.2 vs. 1.6 mm) [38]	No significant difference vs. ball [36, 37]; lower crestal loss than Locator at 10 years (1.6 vs. 2.2 mm) [38]	RCT, meta-analysis, retrospective
Posterior RRR	Limited data	Significantly lower at 10 years (0.9 mm) [38]	Higher at 10 years (1.6 mm) [38]	Retrospective [38]
5-year implant survival	100% [36]	78.2% prosthetic success [39]	100% [36]	RCT [36, 39]
Maintenance burden	Highest (195 events/5 yr) [39]	Intermediate (31 events/5 yr) [39]	Higher complications (1.0/yr) [36]	RCT [36, 39]
Splinting mode	Non-splinted	Non-splinted	Splinted	Meta-analysis: no difference in MBL [37, 42]
Preferred indications	Limited inter-arch space, parallel implants [43]	Routine first-line, stress interruption needed [43]	Severe bone atrophy, need cross-arch stability [43]	Systematic review [43]

FEA: finite element analysis; RRR: residual ridge resorption; MBL: marginal bone loss.

### Occlusal and prosthetic design factors

Reducing cusp inclination and using linear occlusion or lingualised occlusion can reduce lateral forces and bone stress by 19.0%–63.8%, substantially decreasing the load on the residual ridge [44, 45]. When the opposing dentition is a conventional complete denture, bilateral balanced occlusion is recommended to maintain the peripheral seal. In contrast, canine-guided occlusion with an implant-supported fixed prosthesis as the opposing dentition can considerably reduce horizontal torque and peri-implant stress [28, 46].

Under oblique loading, cortical bone stress around implants is considerably greater than that under vertical loading. Distal implants are more prone to stress overload and mechanical complications. Therefore, the anterior–posterior (A–P) spread should be optimised, and cantilever length should be controlled to avoid localised stress injury [32]. Appropriate denture-base extension and adequate relief over pressure-sensitive areas can prevent mucosal ischaemia and bone resorption caused by localised high pressure.

### Patient-related factors

Mucosal thickness and elasticity directly affect pressure distribution. Thin mucosa with poor elasticity is more prone to localised high pressure and accelerated bone resorption. For each 1-mm increase in initial mandibular residual ridge height, annual posterior ridge resorption decreases by approximately 1%. Lower initial bone density is associated with faster and more severe bone resorption [47, 48].

Risk factors for rapid RRR can be stratified into low-, moderate- and high-risk categories. Patients at low risk are systemically healthy, do not smoke, do not wear dentures at night and have favourable mucosal conditions. Patients at moderate risk are aged > 65 years, smoke occasionally or have relatively thin mucosa. Patients at high risk have osteoporosis, diabetes, heavy smoking, long-term nocturnal denture wearing or severe bone resorption [49]. Good compliance, regular follow-up, smoking cessation and systemic health management can substantially improve the bone-preserving effects of IODs [50].

## Current controversies and interpretation

Studies have reported that, following IOD rehabilitation, the rate of mandibular posterior ridge resorption may be slightly higher than that observed with conventional complete dentures. For example, prospective and retrospective radiological studies with extended follow-up durations ranging from 5 to 10 years have documented greater posterior bone loss in IOD groups [51, 52]. However, these findings must be critically interpreted in the context of their specific methodologies. These studies frequently involved relatively small sample sizes and relied on two-dimensional panoramic or cephalometric radiographs, which carry inherent risks of measurement distortion, magnification errors and positioning inconsistencies compared with modern three-dimensional imaging. The main explanations for the accelerated resorption proposed in the literature include excessive occlusal load when the opposing dentition consists of natural teeth or implant-supported fixed prostheses, overly strong attachment retention, excessive cantilever length, insufficient occlusal adjustment and baseline differences in patients’ bone volume [51, 52]. These findings indicate that the bone- preserving effect of IODs is not absolute but is highly dependent on rational design and standardised maintenance [53].

Practical clinical strategies include (1) using low-cusp-inclination occlusal designs with uniform contacts to reduce non-physiological loading, (2) prioritising stress-breaking attachments such as Locator and magnetic systems, (3) strictly controlling cantilever length and optimising the A–P spread, (4) prohibiting nocturnal denture wearing in patients at high risk and (5) regularly reviewing occlusion and attachment wear and performing timely adjustment. These strategies can effectively avoid stress overload and help explain or reduce inconsistencies among study results.

## Advances in digital technologies

In recent years, CBCT three-dimensional reconstruction, computer-guided implant surgery, digital impressions and digital occlusal analysis have increasingly been applied to the precision design of edentulous IOD rehabilitation [54–56]. Digital technologies support precision design and stress control throughout the workflow. CBCT three-dimensional reconstruction can accurately quantify bone volume and key anatomical structures, providing a reliable basis for implant site planning. Computer-guided surgery can improve the accuracy of implant position, angulation and depth; minimise surgical trauma; and optimise stress distribution at the bone interface. Compared with conventional freehand implant placement, guided implant surgery can reduce site deviation by more than 60% and may lower the risk of peri-implant bone resorption [54].

Digital impressions and computer-aided prosthetic design can improve passive adaptation between the denture base and mucosa, distribute occlusal forces more evenly and avoid peripheral extension errors and localised pressure concentration associated with conventional manual impressions. Digital occlusal analysis can quantify occlusal contacts and force distribution, allowing individualised occlusal adjustment and control of prosthetic occlusal error within 20 micrometres [55]. Digital strategies such as tilted implants and the All-on-4^®^ concept can maximise the use of residual bone and avoid complex bone grafting procedures. In patients with severely resorbed edentulous jaws, this approach has achieved 5-year implant survival rates above 95% and better bone preservation than implant placement following traditional bone grafting [56].

At present, digital technology has been integrated into the full IOD workflow, from preoperative planning to postoperative prosthetic delivery. Its core advantages include high precision, reduced trauma and more predictable bone-preserving effects. Limitations include high equipment costs, the need for clinicians to have strong digital competence and the requirement for clinical experience to adjust digital designs under extreme anatomical conditions.

## Clinical implications and stratified recommendations

### Stratified recommendations according to residual ridge bone volume

Clinicians should select IOD protocols according to the bone volume and degree of resorption of the maxillary and mandibular residual ridges. It is important to clarify that although the recommendation for a two-implant mandibular overdenture is an evidence-based conclusion supported by high-level systematic reviews and international consensus, several of the specific stratified recommendations detailed below represent expert opinion derived from biomechanical principles and current clinical observations [57]. For patients with mild-to-moderate mandibular bone resorption, the standard protocol of two implants combined with Locator attachments is preferred as a strongly supported clinical baseline. This approach can control treatment costs while providing satisfactory retention and bone preservation.

For patients with severe mandibular bone resorption, increasing the number of implants to four and combining them with Locator or magnetic attachments is recommended to improve prosthetic stability and reduce localised stress concentration. Considering the generally lower bone density of the maxilla, four or more implants are recommended in this area regardless of bone volume. In cases of severe maxillary bone deficiency, tilted implants or the All-on-4 strategy may be used to maximise residual bone utilisation, avoid complex bone grafting and achieve both functional rehabilitation and bone preservation.

### Stratified recommendations according to systemic health status

Individualised prosthetic design and follow-up management should consider systemic disease, age and adverse habits. Patients in good systemic health without systemic disease may receive conventional IOD treatment and annual follow-up. For patients aged >70 years and those with osteoporosis or diabetes, stress-breaking attachments such as Locator or magnetic systems should be preferred. Follow-up intervals should be shortened to every 3–6 months, and multidisciplinary health management should be conducted with physicians to strictly control systemic risk factors that may interfere with bone remodelling.

For smokers, smoking cessation intervention is required before rehabilitation. The healing period before loading should be appropriately extended, and postoperative oral hygiene education and denture maintenance should be reinforced to reduce the risk of bone resorption and peri-implantitis.

### Stratified recommendations according to economic capacity

Clinical decision-making should consider patients’ economic capacity while ensuring rehabilitation effectiveness and bone preservation. For patients with limited financial resources, a basic treatment protocol consisting of two mandibular implants combined with Locator attachments may be used. This protocol is consistent with international consensus recommendations, involves limited surgical trauma, has relatively low cost and can meet basic requirements for masticatory function and bone preservation.

For patients with adequate financial resources who seek a better long-term prognosis and greater comfort, an optimised treatment protocol is recommended. This may include four implants in both jaws, computer-guided implant surgery, digital impressions, digital occlusal analysis and resilient stress-buffering attachments. Such a fully digital workflow can further improve the uniformity of stress distribution, prosthetic precision and long-term preservation of bone volume.

### Resolving conflicting clinical criteria

Although the aforementioned stratified recommendations provide general baseline guidance, clinical reality frequently presents conflicting criteria. A common scenario is a patient with high-risk factors (e.g. severe posterior bone resorption, systemic comorbidities or advanced age) who has limited financial resources and can only afford the basic two-implant mandibular protocol. In such multidimensional conflicts, a compromised but safe approach must prioritise biomechanical protection over maximum prosthetic retention [58].

When standard multi-implant rehabilitation is economically unfeasible, clinicians must implement strict biomechanical compensation strategies. These include selecting the most resilient stress-breaking attachments (e.g. low-profile Locator or magnetic systems) to minimise non-axial torque on the implants, utilising a lingualised or linear occlusal scheme to reduce lateral shear forces and strictly prohibiting nocturnal denture wear to allow mucosal tissue recovery. Furthermore, the economic savings from the surgical phase should be partially reallocated to a stricter maintenance schedule. Follow-up intervals should be shortened to every 3–4 months to rigorously monitor attachment wear, perform timely relining and evaluate mucosal health. This compensation strategy ensures that, even under financial constraints, patients at high risk can safely benefit from IOD rehabilitation without accelerating RRR.

## Conclusions and future perspectives

IODs demonstrate the capacity to reconstruct physiological stress distribution and regulate inflammatory–molecular signalling pathways, thereby considerably slowing RRR in patients with edentulism and optimising bone remodelling patterns. They represent an ideal rehabilitation approach that balances function, aesthetics and bone preservation.

The bone-preserving effect of IODs is jointly determined by implant design, attachment type, occlusal scheme, soft-tissue conditions and systemic factors. Stress-breaking attachment systems such as Locator attachments may offer advantages in bone preservation under certain clinical conditions. However, current studies still have substantial limitations, including insufficient sample sizes, inconsistent follow-up periods and incomplete elucidation of synergistic mechanisms, as well as an overreliance on finite element analyses. Because computational models simplify complex biological environments, their results must be interpreted with caution and validated by robust clinical data. Consequently, definitive conclusions regarding the absolute superiority of specific attachment systems or treatment protocols should be interpreted cautiously until validated by robust, high-level clinical evidence.

Future research should focus on four directions: (1) conducting large-sample, long-term and multicentre RCTs and meta-analyses to provide higher-level evidence; (2) developing individualised attachment systems and prosthetic designs based on artificial intelligence–assisted stress simulation; (3) establishing IOD-specific optimisation protocols for patients with systemic diseases such as osteoporosis and diabetes; and (4) combining biomaterials that promote osseointegration to further improve bone preservation. This review may provide essential support for more precise and standardised edentulous rehabilitation and improve long-term patient outcomes.

## Data Availability

The data that support the findings of this study are available from the corresponding author upon reasonable request.
